# Efficacy of massage versus massage with post isometric relaxation in temporomandibular disorders: a randomized controlled trial

**DOI:** 10.1186/s13102-024-00865-x

**Published:** 2024-05-16

**Authors:** Mahnoor Tariq, Kainaat Fatima, Shahzada Faiz Ahmad Khan, Wajeeha Mahmood, Tahir Mahmood, Sarma Khurshaid, Masooma Khalid, Mehrunisa Khoosa, Muhammad Naveed Babur

**Affiliations:** 1 Department of Physical therapy & Rehabilitation Sciences, Faculty of Allied health Sciences, Superior University, Lahore, Pakistan; 2 Department of Physical therapy & Rehabilitation Sciences, Faculty of Allied health Sciences, Kainaat Fatima Demonstrator, Superior University, Lahore, Pakistan; 3 Department of Oral & Maxillofacial Surgery, Azra Naheed Dental College, The Superior University, Lahore, Pakistan; 4 Department of Physical therapy, University of Health Sciences, Lahore, Pakistan; 5 Department of Physical Therapy, Rashid Latif Khan University [RLKU] Lahore, Lahore, Pakistan; 6 Physical Therapy Department Jinnah Hospital, Lahore, Pakistan; 7 Physiotherapist Superior University, Lahore, Pakistan; 8 Physical Therapy Department, Allama Iqbal Teaching Hospital, Dera Ghazi Khan, DGK Pakistan; 9 Faculty of allied health Sciences, Superior University, Lahore, Pakistan

**Keywords:** Temporomandibular Joint Disorder, Massage therapy, Post-isometric relaxation exercises, Pain, Maximal mouth opening, Randomized Controlled Trial, Physiotherapy, Masticatory Muscles, Visual Analogue Scale, Therabite Scale

## Abstract

**Background:**

Temporomandibular joint disorder (TMD) is a common condition affecting the masticatory muscles and joint mobility.

**Objectives:**

The primary objective was to compare the effects of massage therapy alone and massage therapy combined with post-isometric relaxation exercises in patients with TMD for pain and maximal mouth opening.

**Design:**

Assessor-blinded randomized controlled trial.

**Setting:**

Sir Ganga Ram Hospital, Chaudhry Muhammad Akram Dental Hospital, Lahore Medical and Dental Hospital.

**Subjects:**

Temporomandibular joint disorder patients.

**Intervention:**

Group A (*n* = 23) received conventional treatment including massage and therapeutic exercises consecutively for 2 weeks. Group B (*n* = 23) received post-isometric relaxation technique along with conventional treatment for consecutive 2 weeks.

**Main measures:**

The main outcome measures were pain and maximal mouth opening. Pain was measured using the Visual Analogue Scale (VAS) and maximal mouth opening (MMO) was measured using the TheraBite Scale.

**Results:**

Both groups demonstrated significant improvements in pain and MMO scores post-treatment. However, Group B (massage with post-isometric relaxation exercises) showed significantly better outcomes compared to Group A (massage alone). There was a statistically significant difference in post-treatment pain scores (*P* = 0.000) and MMO scores (*P* = 0.000) between the two groups.

**Conclusion:**

The results suggest that massage therapy combined with post-isometric relaxation is more effective than massage therapy alone in managing pain and improving mouth opening in TMD patients. The study provides evidence supporting the use of these therapies in TMD management.

**Trial registry number:**

NCT05810831. Date of registration/First submission: 15 March 2023.

**Supplementary Information:**

The online version contains supplementary material available at 10.1186/s13102-024-00865-x.

## Background

Temporomandibular disorders (TMD) are a set of heterogeneous neuromuscular and musculoskeletal diseases affecting a large fraction of the global population outlining multitudinous signs and symptoms including myofascial pain, limited joint mobility, joint noise, and impaired orofacial functions: mandibular deviation, swallowing threshold, chewing, speech, yawing and breathing functions [1, 2]. The prevalence of TMD varies between 7.3 and 30.4% worldwide [3, 4]. A study in Pakistan showed a 40% prevalence of TMD in young adults [5, 6]. The temporomandibular classification is complex as it can be due to masticatory muscles, articular problems, hypermobility syndrome, or displaced disc [7, 8]Myogenic TMD is most common affecting 43.5% of cases of TMD, principally characterized by myofascial pain in the pre-aural area and limited jaw mobility [9–11]. Pain is marked by a reduction in blood flow to muscles and muscular activation leading to metabolism by-product accumulation in muscular tissues, causing fatigue and inflammation [1, 12]. The pain limits joint mobility and decreases maximal mouth opening amplitude thus affecting oral health-related quality of life (OHRQoL) in an unrivaled manner [13, 14]. Over the last few years, the unceasing research on better diagnostic and therapeutic approaches has directed our attention toward the probability of applying non-invasive therapeutic approaches in patients with myogenic TMD [15]. Specifically, the alliance between dentistry and physical therapy helps to settle early diagnosis and substantially enhances the effectiveness and accuracy of therapeutic approaches to follow [16–18]. In physiotherapy management, patient education [19], therapeutic exercises [20] modalities like Transcutaneous Electric Nerve Stimulation TENS [21] Light Amplification by Stimulated Emission of Radiation LASER Therapy [22] and manual therapy [23, 24] are promoted. In the literature, there are various types of research done on therapeutic exercise efficacy in enormous sub-acute and chronic musculoskeletal disorders, and is recommended with a combination of manual therapy (MT) in TMD patients [25]. Therapeutic exercises and manual therapy in PT interventions are used by researchers and clinicians nowadays due to positive outcomes on TMD patients such as decreased pain and improved joint mobility and function of hypotonic muscles [26]. Manual therapy includes soft tissue MT such as myofascial release, post-isometric relaxation (PIR), massage and fascial therapy, and joint MT which includes manipulation [27]. In soft tissue MT, most scientific evidence emphasizes masticatory muscle massage because it improves blood supply and joint range of motion, relaxes muscles, and reduces pain. On the other hand, PIR is a mobilization technique working on phenomena of excitation and relaxation used nowadays in TMD treatment. It reduces muscle tension by inhibiting muscles’ motor neuron field leading to reflex relaxation. The reason is Golgi tendon organs activation when muscles contract. PIR reduces pain, and restores the expected flexibility and length of contracted muscles thus improving joint mobility [7, 28, 29]. Current treatment protocols often rely on a combination of medications, physical therapy, in severe cases, surgery, which may have side effects, are costly, and may not always provide lasting relief while manual therapy has been recognized for its potential benefits in managing TMDs, including pain relief, muscle relaxation, and improved range of motion. However, the comparative effectiveness of massage alone versus massage combined with post-isometric relaxation has not been extensively studied. This research aims to fill this gap in knowledge and provide evidence-based recommendations for TMD treatment. By investigating the comparative efficacy of these interventions, the study could lead to the development of more effective, patient-friendly, and cost-effective TMD therapeutic strategies. The primary objective was to compare the effects of massage therapy alone and massage therapy combined with post-isometric relaxation exercises in patients with TMD for pain and maximal mouth opening. It is hypothesized that there is difference in the effects of massage therapy alone and massage therapy combined with post-isometric relaxation exercises in patients with TMD for pain and maximal mouth opening. Further this difference can be used to identify the superior effects of one therapy over the other for management of pain and mouth opening in TMD.

## Methods

### Trial Design and participants

The study was a single assessor-blinded, parallel assigned randomized controlled trial following CONSORT statement guidelines. The study is registered in Clinicaltrials.gov NCT05810831 and the research was carried out in a manner that was compliant with the laws and regulations that had been set by the ethical committee of Superior University. The trial design was parallel 1:1. The study duration was 6 months after the synopsis approval. 46 TMD patients, both sexes with age 25–45 years and above were recruited from Sir Ganga Ram Hospital, Chaudhry Muhammad Akram Dental Hospital, and Lahore Medical and Dental Hospital from April 2023 to August 2023.

### Group allocation

The participants fulfilling inclusion criteria were randomly assigned to Group A and Group B through a random number table. The allocation process was concealed from the researchers and the participants. The process was completed by a research assistant who did not participate in any further research steps. Outcome assessors who were blinded to the treatment groups were recruited to take pre-treatment and post-treatment level readings. Follow-up was taken after 2 weeks of interventions [7].

### Inclusion and exclusion criteria

All patients in age of 25–45 both genders went through extraoral and intraoral dental examinations carried out by trained dentists in orofacial pain and the patients fulfilling Group I of RDC/TMD criteria were then referred to physiotherapy treatment [7]. Patients with the absence of temporomandibular disc displacement with or without reduction, full dental arches with missing teeth replaced with fixed dental prostheses or full dental arches with natural teeth and good general health (absence of chronic diseases which may affect temporomandibular joint or the muscles of mastication) were included in the study [10] and the patients with earlier splint therapy, injury of masticatory organ, pharmacotherapy (e.g., hormone replacement therapy, oral contraception, and antidepressants), undergoing orthodontic treatment, fibromyalgia and inflammation in the oral cavity (e.g., impacted molars and pulp inflammation) were excluded from the study [7].

### Sample size

**Fig. 1:**
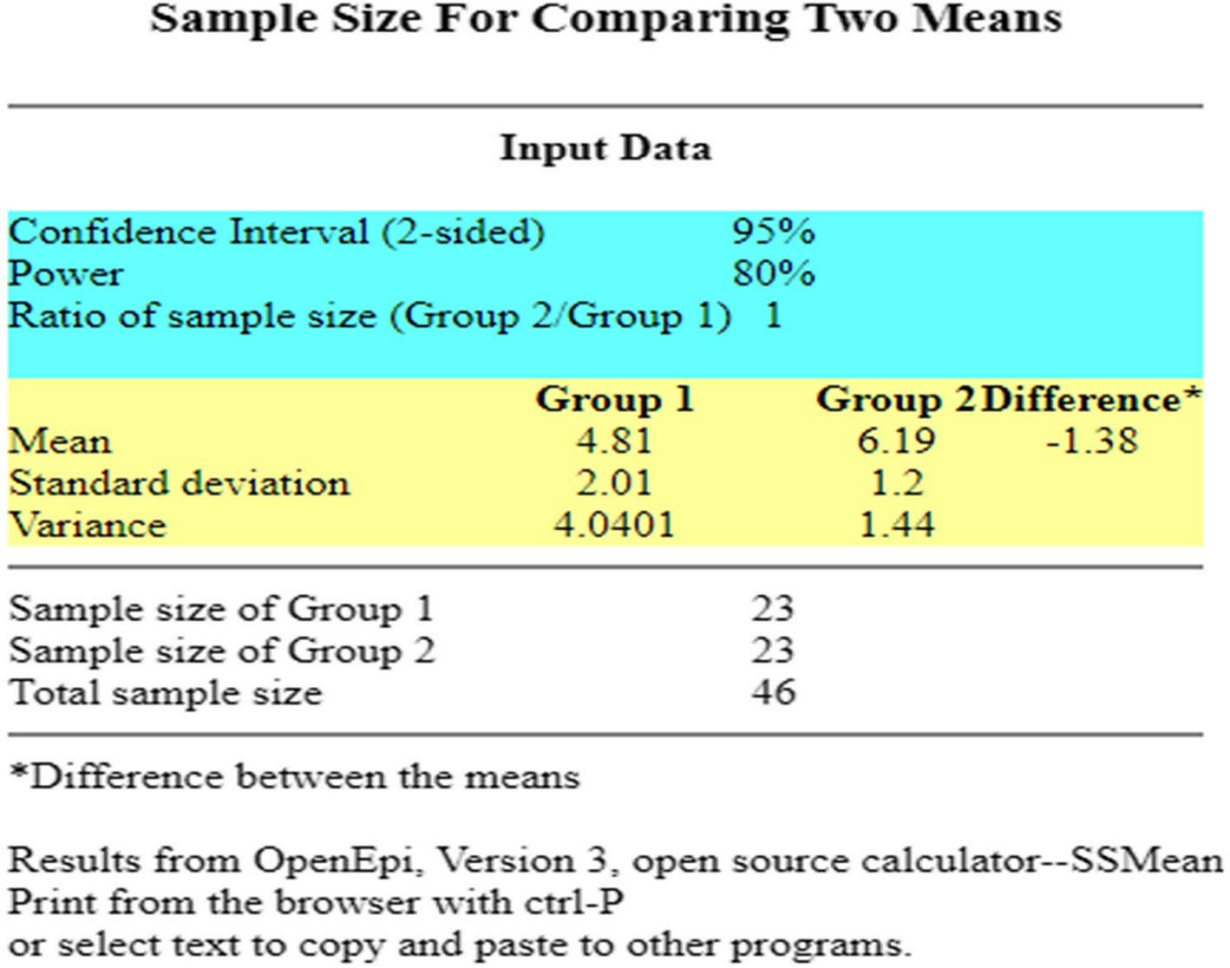
Sample size parameters

The sample size was calculated for this research with a 5% margin of error, 95% level of confidence, 80% of power, and effect size (Cohen D) 0.834, and the ratio of sample size Group A / Group B = 1 using the open Epi tool version 3. The mean value for Group A was 4.81 ± 2.01 and for Group B was 6.19 ± 1.2 and variance was 4.040 and 1.44 respectively [30]. The calculated sample size was 46 with 23 subjects in each group. ***(***Fig. 1***)***

### Outcome measures

The outcome measures were pain and maximal mouth opening [31]. For pain, Visual Analogue Scale was used [1]. The intensity of pain was assessed on a 10 cm horizontal line on the Visual Analogue Scale (VAS) in each patient. The left margin of the scale indicates “no pain” and the right indicates “most imaginable pain”. The scoring is 0–10: 0: no pain. 1–3: mild pain. 4–6: moderate pain. 7–9: severe pain. 10: worst pain [30]. The test-retest reliability of VAS is good (*r* = 0.94) For limited joint mobility maximum mouth opening was measured [31]. The pain-free maximal distance between the incisal edges of the mandibular central incisor and the maxillary central incisor is referred to as the maximal mouth opening and was measured by the Therabite scale. The scoring of the scale ranges from 0 to 60 mm: 0–40 mm: abnormal 40–45 mm: warning 45–60 mm: normal. The reliability of Therabite scale is excellent (*r* = 0.92) [32]. Follow-up was taken after 2 weeks of interventions [7].

### Interventions

#### Group A

In Group A participants received conservative physiotherapy protocol including massage twice during a visit for 2 weeks (excluding Saturdays and Sundays) and an exercise program for 2 weeks, 5 sets of 10 repetitions of each exercise daily. The exercise program included exercises such as Gerry’s exercise, active exercise for mandible’s lateral movement, protrusion, and mouth opening, side-to-side exercise, and active flexion and extension of the cervical spine with instructions on how to perform these exercises were given to each patient. The massage was performed in a supine lying position with a neutrally positioned head. For intraoral massage, the therapist’s hand position was “pincer grip”, thumb placed outside the mouth, and index finger inside the mouth. 10 horizontal movements were carried out from the medial to lateral side of the masseter muscle and 10 vertical movements from the upper to lower side of the muscle. For functional massage, with a pincer grip, the therapist asked the patient to open and close the mouth slowly within the limit of pain and discomfort and performed 10 vertical movements from upper to lower attachment of muscle [7].

#### Group B

In Group B participants received post-isometric relaxation technique 6 times during a visit, 3 times for adductors, and 3 times for lateral movements for 2 weeks (excluding Saturdays and Sundays) [7] in addition to conservative physiotherapy protocol. Post-isometric relaxation was performed in a supine lying position with a neutrally positioned head [10]. To relax the adductors of the mandible, the therapist placed thumbs on the molar and premolar chewing surface of the patient’s mouth and abducted the mandible passively, until a functional barrier was reached. The patient performed adductors isometric contraction for 10s using 20% of maximum force and then relaxed muscles and the therapist abducted the mandible to the new functional barrier. Due to the fact that adductors also contract during lateral mandible movement muscles of the mandible for lateral movement also underwent post isometric relaxation technique. The therapist placed one hand on the mandible and stabilized the patient’s head with the other hand to counterbalance the muscle contraction performed by the patient. Passive lateral translation of the mandible was carried out and the patient performed 10s isometric contraction using 20% of maximum force towards the starting position. Simultaneously, the therapist stabilized the head and balanced the contraction performed by the patient. After contraction, the patient relaxed the muscles and the therapist deepened the lateral movement of the mandible to a new functional barrier [33].

### Data analysis procedure

The SPPS version 20.0 was used for data analysis. The normality was checked using the Shapiro-Wilks test. The *p*-value statistical significance was < 0.05. The data was not normally distributed, so a non-parametric test was used for comparing outcomes pretest and posttest. The within-group analysis was carried out using Wilcoxon signed ranks and the Mann-Whitney U test was used for between-group compassion at *P* value < 0.05 (CI 95%).

## Results

**Fig. 2:**
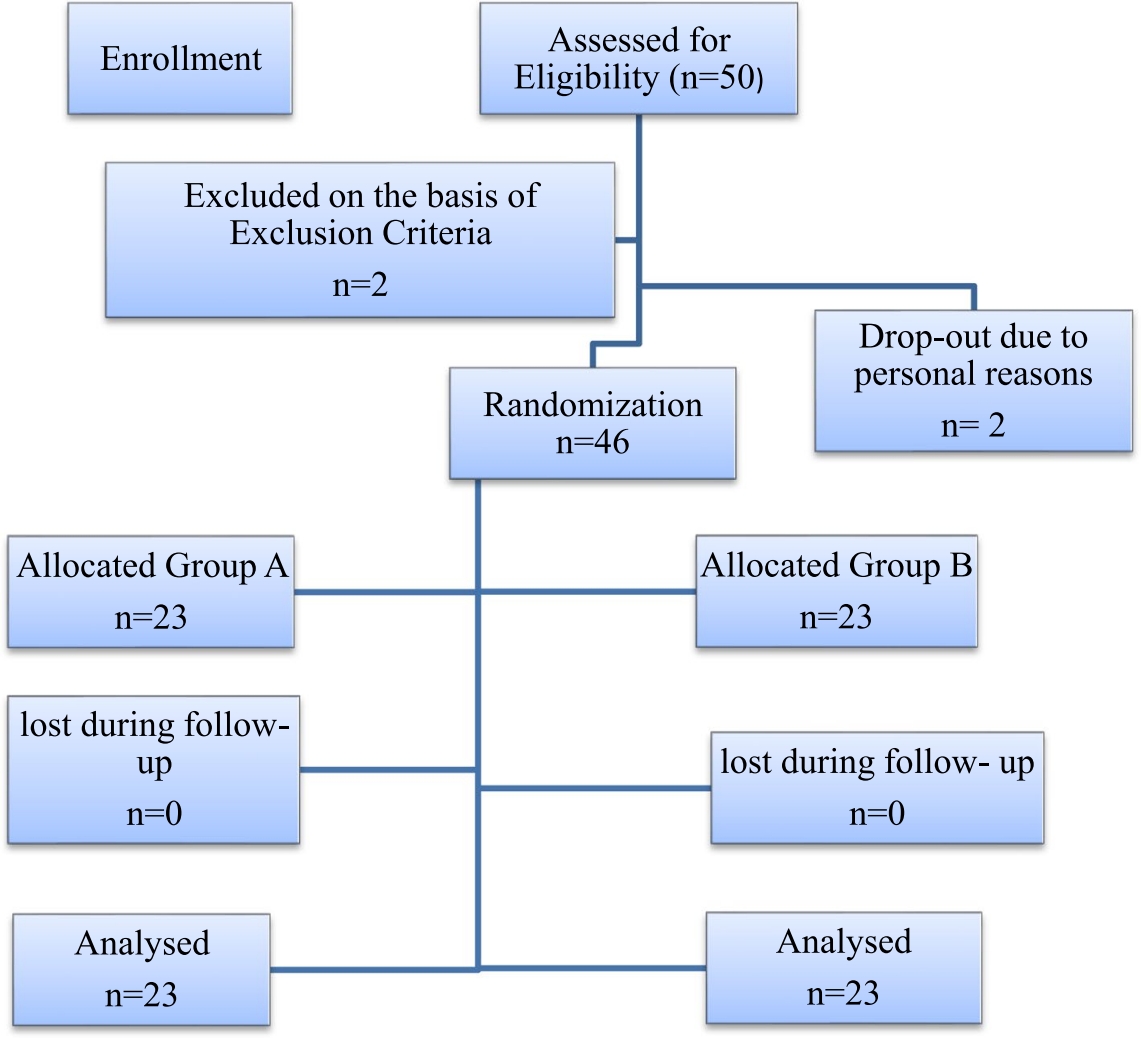
CONSORT flow chart for enrollment, intervention and follow up

During the study period 50 patients were enrolled. 2 were excluded due to ineligibility and 2 moved out due to personal reasons. (Fig. 2**)** Out of 46, there were 23 participants in Group A, 8 were male and 15 were female, while in Group B, out of 23 total patients, 6 were male and 17 were female with a *P* value of 0.522. The mean age of patients in Group A is 36.39 ± 3.57, while in Group B, age is 35.22 ± 2.44. The *p*-value for age was 0.201. The mean duration of TMD, mean pain score, and mean MMO score *p*-value show no statistically significant difference between the two groups. It’s presented in Table 1.

**Table 1:** Demographic profile of participants

Demographics	Group of Treatment	Mean	Std. Deviation	*P* value
**Age of the Patients**	Group A	36.39	3.577	0.201
	Group B	35.22	2.449	
**Duration of temporomandibular disorder (TMD)**	Group A	188.91	28.523	0.407
	Group B	180.87	36.233	
**Pain Score (VAS) Pretest**	Group A	6.22	0.850	0.156
	Group B	5.83	0.984	
**MMO score Pretest**	Group A	35.00	2.256	0.229
	Group B	35.91	2.795	

*P value was significant at < 0.05

Pain score: The pre-intervention score was 6.22 ± 0.850 for Group A and 5.83 ± 0.984 for Group B.

### Within Group Analysis

For Group A, the pre-treatment pain score was 6.22 ± 0.850, which decreased to 3.30 ± 0.559 post-treatment, showing a significant reduction in pain (Δ = 2.92). For Group B, the pre-treatment pain score was 5.83 ± 0.984, which decreased to 2.48 ± 0.511 post-treatment, showing a significant reduction in pain (Δ = 3.35). Both of these changes were statistically significant (*P* = 0.000). It’s presented in Table 2.

**Table 2:** Within Groups Analysis for Pain and MMO (Wilcoxon Signed Rank Test)

Descriptive Statistics										*P*-value
Group of Treatment		N	Mean	Std. Deviation	Minimum	Maximum	Percentiles			
							25th	50th (Median)	75th	
**Group A**	**Pretest**	23	6.22	0.850	5	8	6.00	6.00	7.00	0.000
	**Posttest**	23	3.30	0.559	2	4	3.00	3.00	4.00	
**Group B**	**Pretest**	23	5.83	0.984	4	7	5.00	6.00	7.00	0.000
	**Posttest**	23	2.48	0.511	2	3	2.00	2.00	3.00	

*p-value was significant at < 0.05

Maximal Mouth Opening Score: The pre-intervention score was 35.00 ± 2.256 for Group A and 35.91 ± 2.795 for Group B.

### Within group analysis

For Group A, the pre-treatment MMO score was 35.00 ± 2.256, which increased to 43.30 ± 0.974 post-treatment, showing a significant increase in MMO (Δ = 8.3). For Group B, the pre-treatment MMO score was 35.91 ± 2.795, which increased to 47.04 ± 0.825 post-treatment, showing a significant increase in MMO (Δ = 11.13). Both of these changes were statistically significant (*P* = 0.000). It’s presented in Table 3.

**Table 3:** Within Groups Analysis for MMO (Wilcoxon Signed Rank Test)

Descriptive Statistics										*P*-value	
Group of Treatment		N	Mean	Std. Deviation	Minimum	Maximum	Percentiles				
							25th	50th (Median)	75th		
**Group A**	**Pretest**	23	35.00	2.256	30	37	33.00	36.00	37.00	0.000	
	**Posttest**	23	43.30	0.974	42	45	43.00	43.00	44.00		
**Group B**	**Pretest**	23	35.91	2.795	30	42	34.00	35.00	37.00	0.000	
	**Posttest**	23	47.04	0.825	46	48	46.00	47.00	48.00		

*p-value was significant at < 0.05

### Between-group analysis

The between-group analysis of pain scores and maximal mouth opening (MMO) scores for two groups. Pain mean rank in group A pretest was 25.87 and posttest was 30.91 (*P* < 0.05) compared group B with mean rank of 21.13 and 16.09 pretest posttest respectively with statistically significant (*P* = < 0.05)). While MMO mean rank in group A pretest was 22.00 posttest was 12.00 (*P* < 0.05) and in group B was 25.00 and 35.00 pretest posttest respectively. The difference in pain scores between Group A and Group B post-treatment was statistically significant (P = < 0.05), with a large effect size (Cohen D = 1.53) in group B .Similarly, the MMO score difference post-treatment was statistically significant (*P* = 0.000) with a very large effect size (Cohen D = 4.14) for group B. This suggests that both groups improved with treatment, but Group B received combination (which received massage therapy plus post-isometric relaxation exercises) therapy showed greater improvements in both pain scores and MMO scores post-treatment compared to Group A (which received only massage therapy).It’s presented in Table 4.

**Table 4:** **Between Groups Analysis for Pain and MMO (Mann Whitney U test)**

Outcomes	Measurement	Group A: Massage therapy		Group B: Massage therapy plus post-isometric relaxation exercises		*P*-value
		Mean Rank	Sum of Mean Rank	Mean Rank	Sum of Mean Rank	
**Pain**	**Pretest**	25.87	595.00	21.13	486.00	0.208
	**Posttest**	30.91	711.00	16.09	370.00	0.000
**MMO score**	**Pretest**	22.00	506.00	25.00	575.00	0.437
	**Posttest**	12.00	276.00	35.00	805.00	0.000

*P*-value was significant at < 0.05*

## Discussions

This assessor-blinded randomized controlled trial conducted at Sir Ganga Ram Hospital, Chaudhry Muhammad Akram Dental Hospital, Lahore Medical and Dental Hospital in Temporomandibular disorder patients. The Group A received conventional treatment including massage and therapeutic exercises and Group received post post-isometric relaxation technique. The results suggested that massage therapy combined with post-isometric relaxation is more effective than massage therapy alone in managing pain and improving mouth opening in TMD patients. Another study concluded that post isometric relaxation is good for temporomandibular joint dysfunction, especially in pain reduction. They also suggested that manual techniques have beneficial effects for masticatory muscles and help in release of endorphins that reduce pain [10]. The current study suggested that combination of Physical therapy techniques are effective for TMJ dysfunction. As massage with post isometric relaxation was more beneficial than massage alone in current study. Another study concluded that monotherapy is not effective for TMJ joint dysfunction, as post isometric relaxation with therapeutic exercises and massage with therapeutic exercises were more effective than therapeutic exercises alone [7]. In our study after the intervention, both groups showed a significant reduction in pain and an improvement in MMO. However, the group that received massage combined with post-isometric relaxation exercises (Group B) demonstrated significantly better outcomes than the group that received massage alone (Group A). The post-treatment visual analogue scale (VAS) numeric pain rating scores and Maximum Mouth Opening (MMO) scores were both significantly better in Group B. The distribution of patients across the pain and MMO after treatment also differed but non-significantly between the two groups, with a higher proportion of patients in Group B reporting reduction in pain and achieving normal MMO. Another study concluded that there must be a comprehensive plan for physical therapy treatment rather a single technique [31, 32]. This perspective is same as the current study. Post isometric relaxation is effective for pain and range of motion in TMJ joint dysfunction. Literature supports this concept. Current study showed significant results with post isometric relaxation technique and massage A systematic review suggested that combination of manual therapy with therapeutic exercise is effective for maximal mouth opening and pain in TMJ joint dysfunction. Therapeutic exercises included isometric exercises [12]. This study all supports the concept of current study to use combination of physical therapy techniques for pain and maximal mouth opening in TMJ joint dysfunction. A systematic review concluded that massage is effective for pain in TMJ joint dysfunction [32]. Pessoa and fellows utilized a combination of facial massage, dry needling, and laser therapy in their intervention. Their findings indicated an improvement in temporomandibular disorders, which is consistent with the results of our study. However, our study used a different combination of interventions (massage therapy and post-isometric relaxation exercises), suggesting that multiple intervention strategies can be effective for TMD [1]. Gębska conducted a similar study examining the efficacy of manual soft tissue therapy and therapeutic exercises in patients with pain and limited TMJ mobility. They also reported positive results, aligning with our findings. However, the difference in the therapeutic exercises used in both studies (post-isometric relaxation exercises vs. other therapeutic exercises) underlines the need for further research to ascertain the best combination of techniques [7]. 

This suggests that the addition of post-isometric relaxation exercises to massage therapy may provide additional benefits in managing pain and limited joint mobility in patients with temporomandibular disorders. In current study, massage therapy and massage combined with post-isometric relaxation exercises were effective in reducing pain and improving joint mobility. Prior to the intervention, there were no significant differences between the two groups in terms of age, duration of TMD, gender, initial pain scores, or initial MMO scores. Similarly Herrera-Valenci and colleagues concluded that manual therapy is effective for temporomandibular joint disorders. This supports our findings and confirms that the improvements observed in our study are consistent with a larger body of evidence [25]. Current study concluded that massage with post isometric relaxation in temporomandibular disorders is better than massage alone for pain. Urbański highlighted the importance of manual techniques for muscle relaxation in the treatment of TMD. Our study also used a manual technique (massage therapy), and the results support their assertion, further reinforcing the effectiveness of manual techniques in managing TMD [10]. In contrast, Asquini focused on predictors of pain reduction following manual therapy. While our study didn’t specifically look at predictors, our results do show a significant reduction in pain, thus supporting their thesis that manual therapy is effective [11] Hemashree emphasized conservative treatment modalities in TMD management. Our study used massage therapy and exercises which are conservative treatments, and showed significant improvements, supporting their findings [34]. Penlington focused on psychological therapies for TMDs. Although our study did not investigate psychological therapies, the significant improvements in pain and mouth opening observed in our study indicate that a combination of physical and psychological therapies could potentially offer even greater benefits [35]. Nagata compared the effectiveness of mandibular manipulation and improved multimodal therapy. The results align with our study as both demonstrated a significant improvement in mouth opening. However, our study used massage and exercises instead of mandibular manipulation, suggesting multiple treatment modalities can be effective [23]. Lucena highlighted the effectiveness of manual therapy in older adults. While our study did not specifically focus on older adults, the overall positive results suggest that our intervention could also be effective in this population [26]. 

De Melo conducted a systematic review focusing on manual therapy for myofascial pain related to TMD. The article found it to be effective, which aligns with our results, further validating the use of manual therapy techniques in managing TMD-related pain [29]. Abe investigated the immediate effect of masticatory muscle activity with transcutaneous electrical nerve stimulation in TMD patients. Although our study did not involve electrical stimulation, the positive outcomes in both studies suggest that multiple modalities can contribute to managing TMD effectively [30]. Shousha conducted a randomized controlled trial comparing conservative physiotherapy and occlusive splinting. The study found both methods effective in improving pain and range of motion. This supports our results, adding to the evidence that conservative physical treatments can be effective for TMD [36]. Further it is suggested that combination of physical therapy, psychological therapy and pharmacological methods should be used for comprehensive treatment. These findings suggests that the addition of post-isometric relaxation exercises to massage therapy, the therapeutic effects were better in combination in reducing pain and improving joint mobility temporomandibular disorders. As the statistical analysis revealed a larger effect size in group B compared to group A, showing combined effect of both interventions greater than group A. As Nagata e et al. stated that massage and exercises due to additive effect are more beneficial suggesting multiple treatment modalities can be predominantly effective [23]. 

### Limitations

This study has some limitations. The study did not evaluate the long-term effects of the interventions, restricting our understanding of the sustainability of the observed improvements. Longitudinal studies should be conducted to assess the long-term effects of the interventions. Another limitation is potential confounding factors such as psychological stress, diet, or other health conditions were not considered, which may impact TMD symptoms. Additional research is needed to explore the impact of potential confounding factors on TMD symptoms and the effectiveness of the interventions. Future studies could compare the effectiveness of different combinations of interventions, as multiple treatment modalities appear to be effective for TMD. Consider integrating physical treatments with psychological therapies to potentially offer even greater benefits for TMD patients.

## Conclusion

The findings from this study suggest that massage therapy and post-isometric relaxation exercises are effective in managing pain and improving mouth opening in patients with temporomandibular disorders. Both Group A and Group B showed significant improvements post-treatment in pain scores, MMO scores. However, the group that received massage combined with post-isometric relaxation exercises (Group B) demonstrated significantly better outcomes than the group that received massage alone (Group A).

### Supplementary Information

**Supplementary Material 1.**

**Supplementary Material 2.**

## Data Availability

The data sets used or/and analyzed during this study are available from corresponding authors on a reasonable request.

## Abbreviations

TMD: Temporomandibular Joint Disorder
MMO: Maximum Mouth Opening
VAS: Visual Analogue Scale
RDC/TMD: Research Diagnostic Criteria for Temporomandibular Disorders
SPSS: Statistical Package for the Social Sciences
SD: Standard Deviation
P: P-value, Probability Value
CI: Confidence Interval
TMJ: Temporomandibular Joint
MT: Manual Therapy
PT: Physical Therapy or Physiotherapy
MFP: Myofascial Pain
ROM: Range of Motion

## References

[1] Pessoa DR, Costa DR, Prianti BM, Costa DR, Delpasso CA, Arisawa EÂLS (2018). Association of facial massage, dry needling, and laser therapy in Temporomandibular Disorder: case report.

[2] Ahmed MR, Khalid B, Orakzai GS, Khan RS, Mahmood A, Hassan R (2018). Incidence of temporomandibular disorders among dental students. Age.

[3] Christidis N, Lindström Ndanshau E, Sandberg A, Tsilingaridis G (2019). Prevalence and treatment strategies regarding temporomandibular disorders in children and adolescents—A systematic review. J Oral Rehabil.

[4] Fertout A, Manière-Ezvan A, Lupi L, Ehrmann E (2022). Management of temporomandibular disorders with transcutaneous electrical nerve stimulation: a systematic review. CRANIO®.

[5] Malik W, Malik S, Shakir S, Khan A, Qadeer A, Malik W (2022). Severity patterns of Temporomandibular disorders in young adults with suspected clinical features. Pakistan J Med Health Sci.

[6] Dąbrowska M, Paruszewska-Achtel M, Lisiecki J, Biernacki M, Ulenberg A, Ulenberg G (2019). Rehabilitation in dysfunctions of temporomandibular joints. J Educ Health Sport.

[7] Gębska M, Dalewski B, Pałka Ł, Kołodziej Ł (2023). Evaluation of the efficacy of manual soft tissue therapy and therapeutic exercises in patients with pain and limited mobility TMJ: a randomized control trial (RCT). Head Face Med.

[8] Anandkumar S, Manivasagam M (2022). Physical therapist guided active intervention of chronic temporomandibular disorder presenting as ear pain: a case report. Physiother Theory Pract.

[9] Chan NHY, Ip CK, Li DTS, Leung YY (2022). Diagnosis and treatment of myogenous temporomandibular disorders: a clinical update. Diagnostics.

[10] Urbański P, Trybulec B, Pihut M (2021). The application of manual techniques in masticatory muscles relaxation as adjunctive therapy in the treatment of temporomandibular joint disorders. Int J Environ Res Public Health.

[11] Asquini G, Bianchi AE, Heneghan NR, Rushton AB, Borromeo G, Locatelli M (2019). Predictors of pain reduction following manual therapy in patients with temporomandibular disorders: a protocol for a prospective observational study. BMJ Open.

[12] Arribas-Pascual M, Hernández-Hernández S, Jiménez-Arranz C, Grande-Alonso M, Angulo-Díaz-Parreño S, La Touche R (2023). Effects of physiotherapy on pain and mouth opening in Temporomandibular disorders: an umbrella and mapping systematic review with meta-meta-analysis. J Clin Med.

[13] Crăciun MD, Geman O, Leuciuc FV, Holubiac IŞ, Gheorghiţă D, Filip F (2022). Effectiveness of physiotherapy in the treatment of Temporomandibular joint dysfunction and the relationship with cervical spine. Biomedicines.

[14] Gil-Martínez A, Paris-Alemany A, López-de-Uralde-Villanueva I, La Touche R (2018). Management of pain in patients with temporomandibular disorder (TMD): challenges and solutions. J Pain Res.

[15] ŞAHİN D, MUTLU EK (2021). Physiotherapy interventions in Temporomandibular disorders. Sağlık Profesyonelleri Araştırma Dergisi.

[16] Rocabado M, Gutierrez R, Gutierrez MF, Gutierrez MJ. Case report: Anterior open bite correction treatment by dental treatment and physical therapy through craniocervical mandibular and occlusal stabilization. CRANIO®. 2021:1–6. 10.1080/08869634.2021.2014168. 10.1080/08869634.2021.2014168 34890299

[17] von Piekartz H, Schwiddessen J, Reineke L, Armijo-Olivio S, Bevilaqua-Grossi D, Biasotto Gonzalez DA (2020). International consensus on the most useful assessments used by physical therapists to evaluate patients with temporomandibular disorders: a Delphi study. J Oral Rehabil.

[18] Shahine MS, Mahran SA, Galal MAA-A (2023). Telerehabilitation of Temporomandibular dysfunction syndrome during the COVID-19 pandemic: a pilot study. Egypt J Hosp Med.

[19] Bonato LL, Quinelato V, De Felipe Cordeiro P, De Sousa E, Tesch R, Casado P (2017). Association between temporomandibular disorders and pain in other regions of the body. J Oral Rehabil.

[20] Małgorzata K-M, Małgorzata P, Kinga S, Jerzy S (2021). Temporomandibular joint and cervical spine mobility assessment in the prevention of temporomandibular disorders in children with osteogenesis imperfecta: a pilot study. Int J Environ Res Public Health.

[21] Pihut M, Zarzecka-Francica E, Gala A (2022). Physiotherapeutic rehabilitation of adolescent patients with temporomandibular disorders. Folia Med Cracov.

[22] Xu G-Z, Jia J, Jin L, Li J-H, Wang Z-Y, Cao D-Y (2018). Low-level laser therapy for temporomandibular disorders: a systematic review with meta-analysis. Pain Res Manage..

[23] Nagata K, Hori S, Mizuhashi R, Yokoe T, Atsumi Y, Nagai W (2019). Efficacy of mandibular manipulation technique for temporomandibular disorders patients with mouth opening limitation: a randomized controlled trial for comparison with improved multimodal therapy. J Prosthodontic Res.

[24] Dias WCFGS, Cavalcanti RVA, Júnior Magalhães HV, Pernambuco LA, Alves GÂDS, editors. Effects of photobiomodulation combined with orofacial myofunctional therapy on the quality of life of individuals with temporomandibular disorder. Codas. 2022;34(5):e20200313. 10.1590/2317-1782/20212020313. 10.1590/2317-1782/20212020313 PMC9886175 35416889

[25] Herrera-Valencia A, Ruiz-Muñoz M, Martin-Martin J, Cuesta-Vargas A, González-Sánchez M (2020). Efficacy of manual therapy in temporomandibular joint disorders and its medium-and long-term effects on pain and maximum mouth opening: a systematic review and meta-analysis. J Clin Med.

[26] Lucena LO, Nascimento CMM, Asano NMJ, Coriolano, MdGWdS, Lins CCdSA (2022). Manual therapy for temporomandibular disorder in older adults: an integrative literature review. Revista CEFAC.

[27] Bialosky JE, Beneciuk JM, Bishop MD, Coronado RA, Penza CW, Simon CB (2018). Unraveling the mechanisms of manual therapy: modeling an approach. J Orthop Sports Phys Ther.

[28] Armijo-Olivo S, Pitance L, Singh V, Neto F, Thie N, Michelotti A (2016). Effectiveness of manual therapy and therapeutic exercise for temporomandibular disorders: systematic review and meta-analysis. Phys Ther.

[29] de Melo LA, Bezerra de Medeiros AK, Campos M, Bastos Machado de Resende CM, Barbosa GAS, de Almeida EO (2020). Manual therapy in the treatment of myofascial pain related to temporomandibular disorders: a systematic review. J Oral Facial Pain Headache.

[30] Abe S, Miyagi A, Yoshinaga K, Matsuka Y, Matsumoto F, Uyama E (2020). Immediate effect of masticatory muscle activity with transcutaneous electrical nerve stimulation in muscle pain of temporomandibular disorders patients. J Clin Med.

[31] Cheatham SW, Kolber MJ, Mokha M, Hanney WJ (2018). Concurrent validity of pain scales in individuals with myofascial pain and fibromyalgia. J Bodyw Mov Ther.

[32] Saund DSS, Pearson D, Dietrich T (2012). Reliability and validity of self-assessment of mouth opening: a validation study. BMC Oral Health.

[33] Brighenti N, Battaglino A, Sinatti P, Abuín-Porras V, Sánchez Romero EA, Pedersini P (2023). Effects of an interdisciplinary approach in the management of temporomandibular disorders: a scoping review. Int J Environ Res Public Health.

[34] Hemashree J, Santhosh Kumar M, Chaudhary M (2021). Conservative treatment modalities in the management of temporomandibular joint disorders. Int J Dent Oral Sci.

[35] Penlington C, Bowes C, Taylor G, Otemade AA, Waterhouse P, Durham J (2022). Psychological therapies for temporomandibular disorders (TMDs). Cochrane Database Syst Reviews..

[36] Shousha TM, Soliman ES, Behiry MA (2018). The effect of a short term conservative physiotherapy versus occlusive splinting on pain and range of motion in cases of myogenic temporomandibular joint dysfunction: a randomized controlled trial. J Phys Therapy Sci.

