# Supplementary material for: Efficacy of massage versus massage with post isometric relaxation in temporomandibular disorders: a randomized controlled trial
Source: BMC Sports Sci Med Rehabil. 2024 May 16;16:110. doi: 10.1186/s13102-024-00865-x (PMC11097573; doi:10.1186/s13102-024-00865-x)
Supplement: Supplementary file 2 — Supplementary Material 2. [file 13102_2024_865_MOESM2_ESM.docx]

**Study Protocol**

**Efficacy of massage versus massage with post isometric relaxation in temporomandibular disorders: a randomized control trial**

# **Project Summary:**

Temporomandibular joint disorder (TMD) is a common condition affecting the masticatory muscles and joint mobility. Increasing prevalence of TMD and the need for effective, non-invasive, and easily administered therapeutic interventions have become public health challenge these days. This randomized control trial will be conducted on 46 myogenic TMD patients aged 18 and above. The subjects of this study will be selected fulfilling inclusion criteria and then will be randomized into Group A and Group B. Group A will receive conservative treatment including massage and exercises and Group B will receive post isometric relaxation technique (PIR) in addition with conservative treatment. Pain and Maximal Mouth Opening will be outcome measures and will be assessed at baseline and 2^nd^ week after the treatment.

**General Information about study**

# **Title:**

**Efficacy of massage versus massage with post isometric relaxation in temporomandibular disorders: a randomized control trial**

# **Authors:**

**Mahnoor Tariq^1^, Kainat Fatima^3^, Shahzada Faiz Ahmad Khan, ^3^ Wajeeha Mahmood^4^,Tahir Mahmood^5^, Sarma Khrshaid^6^, Masooma Khalid^7^, Mehrunisa Khoosa,**^8^ **Muhammad Naveed Babur^9^**

**^1^Mahnoor Tariq** Rehab House Officer
Department of Physical therapy & Rehabilitation Sciences, Faculty of Allied health Sciences, Superior University, Lahore Pakistan

Email: [tariqmahnoor150@gmail.com](mailto:tariqmahnoor150@gmail.com)

**^2^ Kainaat Fatima**, Demonstrator Department of Physical therapy & Rehabilitation Sciences
Faculty of Allied health Sciences
Superior University, Lahore Pakistan

Email: [kainaatfatima.pt@gmail.com](mailto:kainaatfatima.pt@gmail.com)

**^3^Shahzada Faiz Ahmad Khan,** Senior Registrar

Department of Oral & Maxillofacial Surgery

Azra Naheed Dental College, The Superior University, Lahore

Email. [ahmad.afghan56@gmail.com](mailto:ahmad.afghan56@gmail.com)
 **^4^Wajeeha Mahmood**, Assistant professor
Department of Physical therapy & Rehabilitation Sciences
Faculty of Allied health Sciences, Superior University, Lahore Pakistan
Email: [higheraimz@hotmail.com](mailto:higheraimz@hotmail.com)
ORCID: <https://orcid.org/0000-0001-9578-583X>

**^5^Tahir Mahmood
Assistant** **Professor**, Rashid Latif College of Physical Therapy [RLCPT]
Rashid Latif Khan University [RLKU] Lahore
Email: [tahirmahmoodphysio@gmail.com](mailto:tahirmahmoodphysio@gmail.com)
Contact: +923006741320
ORCID: <https://orcid.org/0000-0003-0175-52>48

**^6^Sarma Khurshaid** House Officer, Physical Therapy Department
Jinnah Hospital, Lahore Pakistan
Email: [sarmakhushid67@gmail.com](mailto:sarmakhushid67@gmail.com)

**^7^Masooma Khalid**
Physiotherapist, Lahore Pakistan
Email: [kmasooma613@gmail.com](mailto:kmasooma613@gmail.com)

**^8^Mehrunisa Khoosa**
House Officer, Physical Therapy Department , Allama Iqbal Teaching Hospital,
DGK-Pakistan
Email: [mehrunisakhosa200@gmail.com](mailto:mehrunisakhosa200@gmail.com)

**^9^Muhammad Naveed Babur**Professor/Dean, Faculty of allied health Sciences
Superior University, Lahore –Pakistan
[naveed.babur@superior.edu.pk](mailto:naveed.babur@superior.edu.pk)
ORCID: <https://orcid.org/0000-0002-5437-2497>

# **Rationale and Background:**

The rationale for research titled "Efficacy of Massage Versus Massage with Post Isometric Relaxation in Temporomandibular Disorders" stems from the increasing prevalence of Temporomandibular Disorders (TMDs) and the need for effective, non-invasive, and easily administered therapeutic interventions (1). TMDs, characterized by pain and dysfunction in the jaw joint and muscles controlling jaw movement, significantly affect quality of life, limiting daily activities and causing discomfort (2, 3). Current treatment protocols often rely on a combination of medications, physical therapy, and in severe cases, surgery (4). However, these treatments may have side effects, are costly, and may not always provide lasting relief.

Massage therapy and massage with post isometric relaxation technique may be effective in managing TMDs, including pain relief, muscle relaxation, and improved range of motion. However, it is unclear which intervention plan is more effective.

# **Study Objectives:**

We assumed that massage and massage with post isometric relaxation would be effective in myogenic TMD patients for pain and limited joint mobility. So, we aimed to determine the efficacy of massage versus massage with post isometric relaxation in TMD patients.

# **Study Design:**

This single-blinded Randomized Controlled Trial will be conducted at Sir Ganga Ram Hospital, Chaudhry Muhammad Akram Dental Hospital, Lahore Medical and Dental Hospital using parallel groups.

# **Methodology:**

## **Study Population:**

This study will be comprised of 46 myogenic TMD patients.

## **Randomization:**

The participants of this study will be randomly allocated to Group A and Group B using random number table generated by computer program.

## **Study Criteria:**

1. **Inclusion Criteria:**

Following Patients will included in this study: (5, 6)

- Patients with clinical features with myofascial pain and limited mouth opening fulfilling the Axis Group I of RDC/TMD criteria,
- Absence of temporomandibular disc displacement with or without reduction,
- Good general health (absence of chronic diseases which may affect temporomandibular joint or the masticatory muscles),
- Full dental arches with natural teeth or missing teeth replaced with fixed dental prostheses

1. **Exclusion Criteria:**

Following people will be excluded from this study: (7)

- Earlier splint therapy,
- Pharmacotherapy (e.g., hormone replacement therapy, oral contraception and antidepressants),
- Injury of masticatory organ,
- Undergoing orthodontic treatment,
- Inflammation in oral cavity (e.g., impacted molars and pulp inflammation) and fibromyalgia

## **Allocation:**

The allocation process will be concealed through computer generated program where the data will be hidden from the researchers and the participants.

## **Blinding:**

The process of assessment will be completed by a research assistant who will not participate in any further research steps.

23 participants will be enrolled in each group after screening and assessment according to inclusion criteria following CONSORT guidelines.

## **Interventions (Group A and Group B):**

Both groups will receive conservative physiotherapy protocol including massage for 10 days (excluding Saturdays and Sundays). The exercise program with instructions will be given to each patient to perform 5 sets of 10 repetitions daily (7, 8).

**Exercises:**

1. Gerry’s exercise.
2. Active exercise for mandible’s lateral movement.
3. Protrusion and mouth opening.
4. Side to side exercise.
5. Active flexion and extension of cervical spine.

**Massage:**

The massage will be performed in supine position with neutrally positioned head. The treatment will be repeated twice during a visit (7)

1. **Intraoral massage:**

Therapist’s hand position will be “pincer grip” with 10 horizontal movements and 10 vertical movements on masseter muscle.

1. **Functional massage:**

With pincer grip, therapist asked the patient to open and close the mouth slowly within the limit of pain and discomfort and performed 10 vertical movements on masseter muscle.

**Post isometric relaxation:**

Post isometric relaxation will be performed in supine position with neutrally positioned head. The treatment have to repeat 6 times during a visit, 3 times for adductors and 3 times lateral movements (due to fact that adductors also contract during lateral mandible movement) (6). Only Group B received 10 sessions for 10 consecutive days (excluding Saturdays and Sundays) (6, 7):

1. **Relaxing adductors of mandible:**

10secs isometric contraction followed by relaxation.

1. **Relaxing muscles for mandible’s lateral movements:**

10secs isometric contraction followed by relaxation.

# **Safety/ Ethical Considerations:**

The research will be carried out in a manner that will compliant with the laws and regulations that has been set by the ethical committee of Superior University, and the rights of the research participants will be safeguarded.

- Each of the participants will be given their written informed assent.
- Participants will not reveal their identities at any point over the course of the experiment.
- The participants will be informed that there will be no potential drawbacks or dangers associated with the technique of the study.
- It will be made clear to them that they will be free to quit participating in the investigation at any time.
- The information will be protected by a lock and key. It will be safeguarded by a password and will be kept on the portable computer.

# **Outcomes:**

- - - 1. **Pain**

The outcome measures will be pain and maximal mouth opening. The intensity of pain will be assessed on a 10 cm horizontal line Visual Analogue Scale (VAS) in each patient. The left margin of scale indicates “no pain” and right indicates “most imaginable pain”. The scoring is 0-10:

0: no pain. 1-3: mild pain. 4-6: moderate pain. 7-9: severe pain. 10: worst pain (9). The test-retest reliability of VAS is good (r = 0.94) (10).

- - - 1. **Maximum Mouth Opening (MMO)**

For maximal mouth opening inter-incisal distance will be measured on a Therabite scale (11). The scoring of scale ranges from 0 to 60 mm: 0-40 mm: abnormal 40-45 mm: warning 45-60 mm: normal (9). Reliability of Therabite scale is excellent (r = 0.92) (12).

# **Follow up Participants:**

The outcome measures will be pain and maximal mouth opening. These outcomes will be measured at baseline and after 2 weeks of intervention.

# **Data Analysis/ Management:**

The sample size was calculated for this research with 5% margin of error, 95% level of confidence, 80% of power and ratio of sample size Group A / Group B = 1 using open epi tool (13, 14). The participants will be aged 18 and above, will go through extraoral and intraoral dental examinations carried out by trained dentists in orofacial pain and fulfill Group I of RDC/TMD criteria with good general health (5, 6). While patients undergoing orthodontic treatment, pharmacotherapy, injury of masticatory muscles, earlier splint therapy and inflammation in oral cavity will be excluded (7).

For qualitative data, descriptive statistics will be used and for categorical data frequencies will be calculated. The normality of data will be checked and test will be applied accordingly. The P-value of 0.05 (95%) will be considered significant.

# **Duration of Project:**

The duration of study will be from registry of trial on 12^th^ April, 2023 and will be expected to complete on August 30, 2023.

# **Expected study outcomes:**

Massage and massage with post isometric relaxation exercises may be effective to reduce pain and increase maximal mouth opening, however, it is unclear which intervention plan will be most effective.

# **Dissemination of Results/ Publication policy:**

The data sets used or/and analyzed during this study will be available from corresponding authors on a reasonable request. The results will be available to policy makers, health care professionals and health care community after publication in the respective journal but it depends upon the policy of journal.

# **Consort Flow Chart:**

**Problems Anticipated:**

This project will be self-funded and there is still no available source of any funding from any community/ organization or institute.

# **Project –Management / Authors Contribution and responsibility assigned:**

**Mahnoor Tariq** = Conception of work and design of the work.

**Kainaat Imtiaz** = Drafted the work.

**Shahzada Faiz Ahmad Khan =** Examination of patients and revision of work.

**Wajeeha Mahmood** = Acquisition and substantively revised the work

**Tahir Mahmood =** Analysis and interpretation of data.

**Masooma Khalid =** Revised the work.

**Sarma Khurshaid =** Visualization of work.

**Mehrunisa Khosa =** Editing of work.

**Naveed Babur**= Final approval of the submitted version of publication

All authors will approve the final manuscript for submission. It will be confirmed that the content of the manuscript has not been published, or submitted for publication elsewhere following ethical principles of research set by local committee and Helsinki declaration.

# **Informed Consent:**

Informed consent of participants will be obtained before data collection.

# **Budget:**

No any budget available.

# **References:**

1. ŞAHİN D, MUTLU EK, TAŞKIRAN HJSPAD. Physiotherapy Interventions in Temporomandibular Disorders. 2021;3(3):162-70.

2. Pessoa DR, Costa DR, Prianti BdM, Costa DR, Delpasso CA, Arisawa EÂLS, et al., editors. Association of facial massage, dry needling, and laser therapy in Temporomandibular Disorder: case report. Codas; 2018: SciELO Brasil.

3. Ahmed MR, Khalid B, Orakzai GS, Khan RS, Mahmood A, Hassan RJA. Incidence of temporomandibular disorders among dental students. 2018;15:25.

4. Chan NHY, Ip CK, Li DTS, Leung YYJD. Diagnosis and treatment of myogenous temporomandibular disorders: a clinical update. 2022;12(12):2914.

5. Zhang L, Xu L, Wu D, Yu C, Fan S, Cai BJAPM. Effectiveness of exercise therapy versus occlusal splint therapy for the treatment of painful temporomandibular disorders: A systematic review and meta-analysis. 2021;10:6122-32.

6. Urbański P, Trybulec B, Pihut MJIjoer, health p. The application of manual techniques in masticatory muscles relaxation as adjunctive therapy in the treatment of temporomandibular joint disorders. 2021;18(24):12970.

7. Gębska M, Dalewski B, Pałka Ł, Kołodziej Ł. Evaluation of the efficacy of manual soft tissue therapy and therapeutic exercises in patients with pain and limited mobility TMJ: a randomized control trial (RCT). 2022.

8. Bahlool SW, Fakhruddin KS, Hegazy FAJAiB, Sciences H. Effectiveness of cervical spine manual therapy in the management of temporomandibular joint disorders: A systematic review. 2022;1(1):23.

9. Abe S, Miyagi A, Yoshinaga K, Matsuka Y, Matsumoto F, Uyama E, et al. Immediate effect of masticatory muscle activity with transcutaneous electrical nerve stimulation in muscle pain of temporomandibular disorders patients. 2020;9(10):3330.

10. Cheatham SW, Kolber MJ, Mokha M, Hanney WJJJob, therapies m. Concurrent validity of pain scales in individuals with myofascial pain and fibromyalgia. 2018;22(2):355-60.

11. Asquini G, Pitance L, Michelotti A, Falla DJJoor. Effectiveness of manual therapy applied to craniomandibular structures in temporomandibular disorders: A systematic review. 2022;49(4):442-55.

12. Saund DSS, Pearson D, Dietrich TJBoh. Reliability and validity of self-assessment of mouth opening: a validation study. 2012;12:1-4.

13. Khairnar S, Bhate K, SN SK, Kshirsagar K, Jagtap B, Kakodkar PJJoda, et al. Comparative evaluation of low-level laser therapy and ultrasound heat therapy in reducing temporomandibular joint disorder pain. 2019;19(5):289.

14. Kalamir A. Intra-oral myofascial therapy, education and self-care for chronic myogenous temporomandibular disorder: Macquarie University; 2022.
